# Effect of sterilants and plant growth regulators in regenerating commonly used cassava cultivars at the Kenyan coast

**DOI:** 10.1016/j.heliyon.2023.e17263

**Published:** 2023-06-14

**Authors:** Patrick Clay Kidasi, Dora Chao Kilalo, Agnes Wakesho Mwang'ombe

**Affiliations:** a Department of Plant Science and Crop Protection, University of Nairobi, Kenya

**Keywords:** Manihot esculenta, Plant growth regulators (PGRs), Surface sterilization, Shooting, Rooting, Micro propagation

## Abstract

Cassava is an important root crop whose seed system is undeveloped. Micropropagation of explants *in vitro* has the potential of addressing the challenge of the unavailability of healthy cassava planting materials. Therefore, the study determined the effect of sterilization and plant growth regulators on cassava explants to produce certified disease-free plants of commonly used cultivars at the coast of Kenya. The apical nodes drawn from the three cultivars of cassava, Tajirika and Kibandameno and *Taita*, were used as explants. The sterilant sodium hypochlorite (NaOCl) at 5, 10 and 15% and 70% ethanol for 1 and 5 min and sprayed for 20 s were tested for the effect on the explant. Similarly, the effect of BAP (6-Benzyl amino purine) and NAA (1-Naphthalene acetic acid) Plant Growth Regulators (PGRs) each at 0.5, 1 and 5 mg/L under optimal conditions of sterilization was determined. Surface sterilization using 10% NaOCl followed by spraying 70% ethanol for 20 s had 85% initiation on Tajirika whereas 5% NaOCl followed by spraying 70% ethanol for 20 s had 87% and 91% initiation in Kibandameno and *Taita* cultivars, respectively. In Tajirika, significantly (p < 0.05) high shooting of 68% was from 5 mg/L BAP in MS media whereas approximately 50% rooting was from either 0.5 mg/L BAP or 5 mg/L NAA in MS media. Kibandameno and *Taita* cultivars had approximately 50% shooting from MS media without PGRs. Kibandameno had >37% rooting from 0.5 to 5 mg/L BAP or NAA in MS media whereas *Taita* had approximately 50% rooting from 0 to 5 mg/L NAA in MS media. This protocol showed at least 50% success rate of initiation, shooting and rooting as a rapid multiplication regeneration of Tajirika and Kibandameno and *Taita* cultivar plantlets with little modification of humidity and temperatures in the growth chambers. This protocol requires validation for use in large-scale production of cassava plantlets to alleviate the inadequacy of cassava planting materials among farmers.

## 1 Introduction

Cassava (*Manihot esculenta* Crantz) is a climate-smart crop rich in starch content and gluten-free properties. Most developing countries in sub-Saharan Africa are characterized by arid and semi-arid conditions and chronic food challenges, cassava is considered a food and cash crop but its production is limited by viral diseases infections and the unavailability of planting materials [1]. Cassava stem cuttings are in most cases unavailable during planting seasons, therefore, micropropagation is a sustainable approach for rapid multiplication. According to Ref. [2], micropropagation has been vastly incorporated into advancing agriculture, horticulture and industries given its potential of producing healthy seedlings with a reduced vegetal cycle. Of course, micropropagation techniques have a higher multiplication of cassava planting materials compared to conventional cassava cuttings which have a low multiplication potential of ten cuttings per plant per year. Inadequate planting materials immensely contribute to the recycling of cuttings which further disseminate common seed-borne diseases and pests when sourced from infected mother plants. In the case of Kenya, there are several improved cassava cultivars from IITA and KALROs, however, there are scarce (lack of planting materials) or partly not extensively adopted by farmers owing to particular traits preference. Unfortunately, tissue culture technique, especially on most of the cassava landraces, has not been explored for use to purposely mass regenerate cassava planting materials in Kenya.

Sterilization of explants for micropropagation is important in either minimizing or eliminating most of the endogenous and exogenous microorganisms for effective shooting and rooting proliferation. Low initiation is eminent when sterilants are not effective in eliminating all bacterial or fungal microorganisms or damage the explant tissues [3]. For example [4], reported 50% and 13% contaminations on bananas due to bacteria and fungi, respectively. Most bacteria survived in a spore-form which is metabolically inactive but potentially germinates into vegetative cells in favourable conditions. Given the potential of microorganisms in limiting the growth and development of micropropagated explants, several protocols optimized surface sterilization of micropropagation of cassava. An example of a sterilant is sodium hypochlorite which had been reported by Ref. [5] to exert metabolic reactions such as phospholipids destruction fatty acid degradation and chloramine formation against bacteria [5].

Shooting and rooting proliferation in micropropagation are based on the totipotency potential, however, incorporation of plant growth regulators – cytokinins and auxins – have been shown in most studies to be metabolically enhanced. Shoot stimulation and elongation follows due to the potential of cytokinins such as 6-Benzylaminopurine (BAP) to break axillary and bud dormancy by mechanistically triggering cell division and lateral bud development [6,7]. Similarly, auxins such as 1-Naphthalene acetic acid (NAA), in most studies were shown to significantly enhance rooting initiation, growth and development [[8], [9], [10]]. Concentrations of 0.01–10 mg/L NAA were reported by Ref. [11] as the most common. However, for cassava, the best shooting was on MS medium without exogenous auxins [12]. Nodal culture is considered the safest micropropagation technique following the production of true-to-type plants, however, stresses from the exogenous addition of plant growth regulators were shown to induce somaclonal variations as compared to those recovered from field cuttings [13,14].

The varietal difference among plants, especially in cassava had constantly forced on optimization of tissue culture protocols for different purposes [15]. Following the establishment of a lack of cassava planting materials [16] and perpetually infection by viral disease [17], there is a need to explore rapid multiplication techniques of cassava such as tissue culture. There are previous studies that consider the use of at least two sterilants effective for micropropagation to guarantee initiation [18]. However, the use of at least two sterilants on cassava cultivars, especially on landraces, had least been explored. On the other hand, the PGRs had been extensively optimized to enhance shooting and rooting in cassava but with little emphasis on the time taken. For mass propagation, this study investigates optimal surface sterilization and MS media supplementation of PGRs composition to at least induce 50% initiation, shooting and rooting percentage on Tajirika, Kibandameno and *Taita* cultivars. Successful results of this study findings would complement other molecular techniques and rapid multiplication techniques to enhance the availability of clean healthy certified cassava planting materials for smallholder resource-poor farmers.

## 2 Materials and methods

### 2.1 Cassava cultivars and the explant used

Tajirika and Kibandameno and *Taita* landraces were cassava cultivars used for tissue culture. These are popularly grown in Kilifi and Taita Taveta counties of Kenya. Tajirika is an improved cultivar produced by KALRO whereas Kibandameno and *Taita* are landrace cultivars. Cuttings of each cultivar were raised at Kabete Field Station for sourcing ex-plants for tissue culture. The explant was apical nodes of young shoots. During the collection of the ex-plants, the aseptic measures ensured were wearing latex gloves, use of separate surgical blades for the cultivars and surface sterilization before cutting another explant.

### 2.2 Surface sterilization of explants

The explants were aseptically cut and reduced into optimal sizes of 1–2 cm with one node for culturing in the sterilized apparatus. Each cultivar's explants were dipped in a soap detergent for 1 min and thoroughly washed with running tap water until the foam cleared. Thereafter, explants were placed in a clean beaker with distilled water where 2–3 drops of Tween 20 were added. Herein, the explants were thoroughly shaken using a magnetic stirring plate at 1500 rpm for 10 min and rinsed severally using distilled water until the foam cleared. The explants were surface sterilized with NaOCl at 0, 5, 10 and 15% concentrations that were prepared from an absolute solution [Assy (w/w)-99.5%] as treatments. Each of the NaOCl concentrations with explants in separate glass beakers were stirred on a magnetic stirring plate at 1500 rpm for 7 min and rinsed thrice by shaking them in clean beakers using distilled water. Further surface sterilization of explants using 70% ethanol prepared from the absolute solution (99.5% ethanol) was done in a laminar flow hood under fluorescent light with the fans running at a medium speed. The treatments consisted of exposing the rinsed explants to 70% ethanol for periods of 1 min, 5 min and spraying for 20 s before rinsing further with distilled water again. The control treatment was not exposed to 70% ethanol. This experiment was repeated three times for all the cassava cultivars.

### 2.3 Media preparation and supplementation with BAP and NAA hormones

A commercial Murashige and Skoog (MS) media were supplemented with 20 g sucrose, 8 g agar-agar and varying concentrations of Plant Growth Regulators (PGRs) BAP and NAA, separately. Therefore, 4.4 g MS were incorporated with 20 g sucrose in three-quarter liters of distilled water and stirred on a magnetic stirring plate at 1500 rpm for 10 min. The solution was adjusted to a pH of 5.7–5.8 on a digital pH meter using either Sulphuric (V) acid or Sodium chloride before being topped to make a liter of the solution. Thereafter, 8 g agar-agar was added to the solution and melted on a heating and magnetic stirring plate (1500 rpms; 100 °C) for 15 min.

The Plant Growth Regulators, BAP and NAA, each made to a stock solution of 5 mg/L (*w/v*) were separately added in the MS media at concentrations of 0.5, 1 and 5 mg/L. Each test tube for incubation had 5 mls of MS media dispensed with and without plant growth regulators in respective treatments and sealed with a double aluminium foil. The test tubes with media were autoclaved at 121 °C for 20 min (15 psi).

### 2.4 Culturing and incubation of explants

Prepared explants for each treatment were inoculated in test tubes within a laminar flow hood with the fans running at full speed. Cool media from the autoclave was opened one at a time to culture the explants. The forceps were surface-sterilized by dipping in absolute ethanol and flamed and used to pick and culture the explants whereby a quarter of its length was in the media and the nodes facing upwards. Test tube cultures were resealed with the double layer of aluminium foil and reinforced with a cling film followed by their labelling. All test tubes were maintained at 28 ± 2 °C with a cool white fluorescent light on a 16:8-h light photoperiod.

### 2.5 Experimental layout

Each experiment was arranged in a complete randomized design with three replications that were repeated three times. There were 16 treatments (4 × 4 factorial) for NaOCl (5, 10 and 15%), 70% ethanol (1 and 5 min and spray for 20 s), and their combinations and controls as indicated in Table 1. The BAP and NAA each had four treatments that were 0, 0.5, 1, and 5 mg/L. In all the treatments, each replication had 20 test tubes with explants for experimentation.

**Table 1:** Effect of surface sterilization with Sodium hypochlorite (NaOCl) and 70% Ethanol on the initiation (%) of ex-plants of the three cassava cultivars used.

Treatment	NaOCl concentration (%)	70% ethanol duration	Initiation percentage after 14 days		
			Tajirika	Kibandameno	*Taita*
1	0	0	8.33^ab^	11.11^ab^	9.44^abc^
2	0	1	6.66^ab^	15.56^ab^	1.11^a^
3	0	5	1.67^a^	7.22^a^	2.22^ab^
4	0	Spray	6.11^ab^	16.67^ab^	12.78^bcd^
5	5	0	25.55^cd^	38.89^c^	35.55^e^
6	5	1	52.22^e^	47.22^c^	47.78^fg^
7	5	5	31.67^d^	18.33^ab^	14.44^cd^
8	5	Spray	65.55^f^	86.67^d^	90.56^h^
9	10	0	26.11^cd^	41.67^c^	37.22^ef^
10	10	1	47.78^e^	46.11^c^	51.66^g^
11	10	5	30.55^d^	12.78^ab^	7.22^abc^
12	10	Spray	85^g^	53.89^c^	52.78^g^
13	15	0	30.56^d^	38.89^c^	38.33^ef^
14	15	1	46.67^e^	22.78^b^	22.22^d^
15	15	5	15.56^bc^	6.11^a^	1.11^a^
16	15	Spray	55e^f^	46.67^c^	47.78^fg^
**CV**			0.3	10.4	13.6
**L.S.D**			12.96	15.39	11.59

NOTE: “Spray” involved the application of 70% ethanol sparingly by spraying the ex-plants for less than 20 s and rinsing thrice using distilled water. Means in the same column not having a common letter are significantly different (P < 0.05).

### 2.6 Data collection

Initiated explants for each cultivar were determined after 14 days after culturing. Daily records were made on the number of days and the total number of shoots and roots to assess the effect of BAP and NAA on the shooting and rooting potential of the cassava cultivars. These observations continued until no further shooting or rooting was recorded.

In a separate experiment, the number of leaves and the length of shoots were evaluated among five randomly selected explants in each replication of the treatments every week. Among the randomly sampled explants, three explants in each replication were further randomly selected for destructive sampling to determine the roots and root length. Every week, the explants were carefully cleaned off the media with gently running water and the length and number of all roots were determined.

### 2.7 Data analysis

All the experiments were repeated three times and values were presented as means. Statistical analysis was performed using GenStat software (15th Edition). A general ANOVA test with Tukey multiple comparisons was applied to determine statistical significance at P < 0.05 level.

## 3 Results

### 3.1 Effect of NaOCl and 70% ethanol on surface sterilization of cassava cultivars explants

Surface sterilants – Sodium hypochlorite (NaOCl) and 70% Ethanol – at different concentrations and exposure times exerted significant differences in the initiation percentage of Tajirika, Kibandameno and *Taita* cultivars after 14 days. Tajirika cultivar had 25–30% explants initiation after sterilization with NaOCl at a concentration between 5 and 15%. Explants sterilization with 70% ethanol for 1 and 5 min and spraying for 30 s had less than 7% initiation of Tajirika and was not significantly different to those recorded in the control. There was a 50% initiation of Tajirika when sterilized with NaOCl concentrations between 5 and 15%, all followed by 1 min exposure to 70% ethanol. Over 50% initiation of Tajirika was recorded in 5–15% NaOCl, all followed by spraying 70% ethanol for 30 s. Significantly (p < 0.05) highest initiation of 85% in Tajirika was recorded at 10% NaOCl followed by spraying 70% ethanol for 30 s (Table 1).

Kibandameno explants separately sterilized by 5, 10 and 15% NaOCl had 40% initiation which was significantly (p < 0.05) higher than the control. The exposure of Kibandameno explants to 70% ethanol for 1–5 min or spraying for 30 s had 7–17% initiation which was not significantly different from the control. Kibandameno explants sterilized by 5 and 10% NaOCl, all followed by 1 min exposure to 70% ethanol had 47% initiation. Kibandameno explants that were separately sterilized by 5, 10 and 15% NaOCl and exposed to 70% ethanol for 5 min had 6–19% initiation which was not significantly different to the control. There was about 50% initiation of Kibandameno explants separately sterilized by 10 and 15% NaOCl and all followed by spraying 70% ethanol for 30 s. Significantly (p < 0.05) highest Kibandameno explants initiation of 87% was recorded in 5% NaOCl followed by spraying 70% ethanol for 30 s (Table 1).

Sterilization of *Taita* explants using 5, 10 and 15% NaOCl had 36–38% initiation which was significantly (p < 0.05) higher than the control. All the exposure durations of 70% ethanol to *Taita* explants had >13% initiation, statistically similar to that of the controls. About 50% initiation of *Taita* explants was recorded after sterilization with 5 and 10% NaOCl followed by 1 min exposure to 70% ethanol. Use of 5 and 10% NaOCl all followed by 5 min of exposure to 70% ethanol had 7–14% initiation of *Taita* explants, statistically similar to that of the control. Sterilization of *Taita* explants using 10 and 15% NaOCl followed by spraying 70% ethanol for 30 s had about 50% initiation. Significantly (p < 0.05) highest initiation of 90% was recorded on *Taita* explants sterilized using 5% NaOCl followed by spraying 70% ethanol for 30 s (Table 1).

### 3.2 Effect of BAP and NAA on the shooting of three cassava cultivars used

**Fig. 1:**
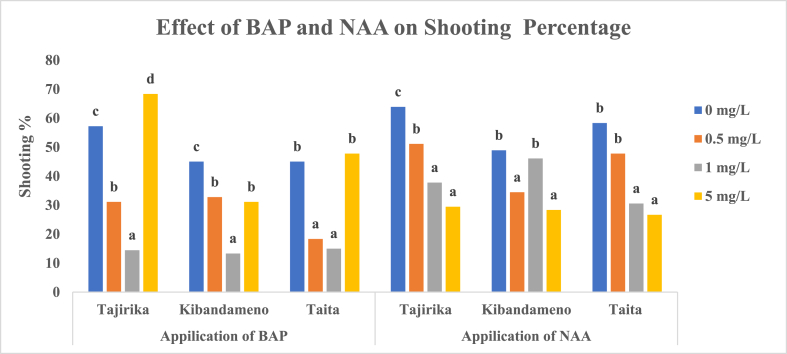
Effect of BAP and NAA in MS media on the shooting percentage of Tajirika, Kibandameno and Taita cultivars. Means in the same variety's bars having the same letter are not significantly different at P < 0.05.

**Fig. 2:**
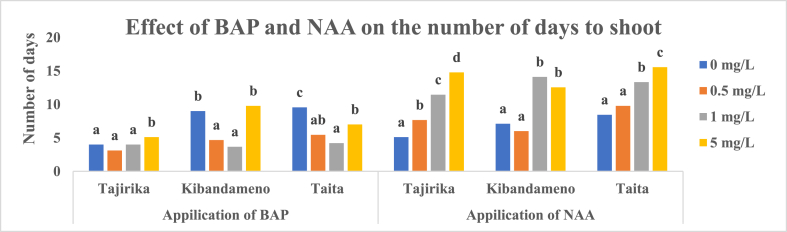
Effect of BAP and NAA in MS media on the number of days to shoot for Tajirika, Kibandameno and Taita cultivars. Means in the same variety's bars having the same letter are not significantly different at P < 0.05.

Before culturing explants to MS media supplemented with either BAP or NAA, Tajirika was surface sterilized in 10% NaOCl and sprayed with 70% ethanol for 20 s while Kibandameno and *Taita* cultivars were surface sterilized in 5% NaOCl and sprayed with 70% ethanol for 20 s. A 50% shooting each in Tajirika and *Taita* explants was recorded when cultured in MS media supplemented with 5 mg/L of BAP. Supplementing MS media with 0.5 and 1 mg/L of BAP had >30% shooting of Tajirika and *Taita* explants. In Kibandameno, significantly (p < 0.05) highest shooting of 45% was recorded in explants cultured in MS media alone. Significantly (p < 0.05) least shooting percent in the three cassava cultivars was recorded in MS media supplemented with either 0.5 or 1 mg/L of BAP, fortunately, used a few 3–5 days to shoot. On the other hand, MS media supplemented with either 0 or 5 mg/L of BAP had significantly (p < 0.05) the highest shooting percentage but took 4–9 days to shoot in the three cultivars (Fig. 1, Fig. 2).

In Tajirika and *Taita* explant cultivars, the addition of 1 and 5 mg/L NAA had significantly (p < 0.05) lower shooting of about 30% whereas MS media alone had significantly (p < 0.05) highest shooting of about 60%. About 50% shooting was recorded in Kibandameno explants cultured in MS media supplemented with 0 and 1 mg/L of NAA and was significantly (p < 0.05) higher than a 30% shooting recorded in MS media supplemented with 0.5 and 5 mg/L of NAA. An increase in NAA from 0 to 5 mg/L in MS media significantly (p < 0.05) increase the number of days to shoot from 5 to 15 days in all the cassava cultivars used. Among the treatments with NAA added into MS media, It was only MS media supplemented with 0.5 mg/L of NAA that had about 50% shooting and took fewer days 7–9 days in Tajirika and *Taita* cultivars (Fig. 1, Fig. 2).

### 3.3 Effect of BAP and NAA on the number of leaves and shoot length of cassava cultivars

**Fig. 3:**
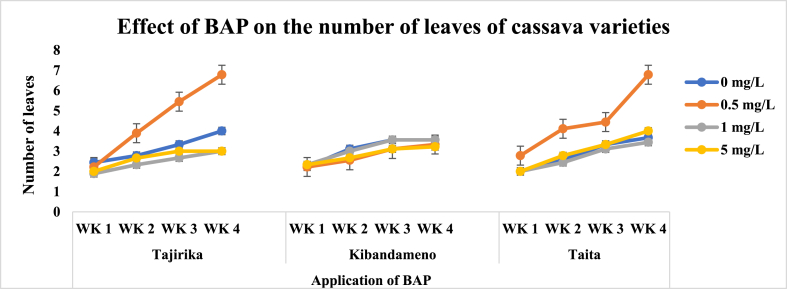
Effect of application of BAP in MS media on the number of leaves of Tajirika, Kibandameno and Taita cultivars. WK represents Weeks.

**Fig. 4:**
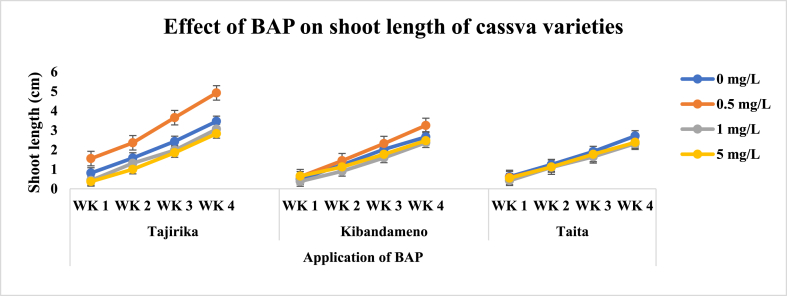
Effect of application of BAP in MS media on the shoot length of Tajirika, Kibandameno and Taita cultivars. WK represent Weeks.

Across all three cassava cultivars, there was a progressive increase in shoot length and number of leaves in the four weeks for the explants cultured in MS media with or without PGRs (Picture 1). In the third and fourth weeks, Tajirika explants cultured in MS media supplemented with 0.5 mg/L of BAP had significantly (p < 0.05) twice more leaves than other treatments. An increase of BAP from 0.5 to 5 mg/L in MS media decreases the shoot length of explants cultured from the first to the fourth week. For instance, MS media supplemented with 0.5 mg/L of BAP had significantly (p < 0.05) the longest shoot length. Kibandameno explants cultured in MS media with or without BAP had a significant (p < 0.05) influence on the shoot length but not on the number of leaves. Significantly (p < 0.05) highest shoot length was recorded in MS media supplemented with 0.5 mg/L of BAP whereas the least was when supplemented with 1 mg/L of BAP. The *Taita* cultivar explants cultured in MS supplemented with BAP had a significant (p < 0.05) increase in the number of leaves but not in the shoot length. Significantly (p < 0.05) highest number of leaves were recorded in *Taita* cultivar explants cultured in MS media supplemented with 0.5 mg/L of BAP (Fig. 3, Fig. 4).

**Fig. 5:**
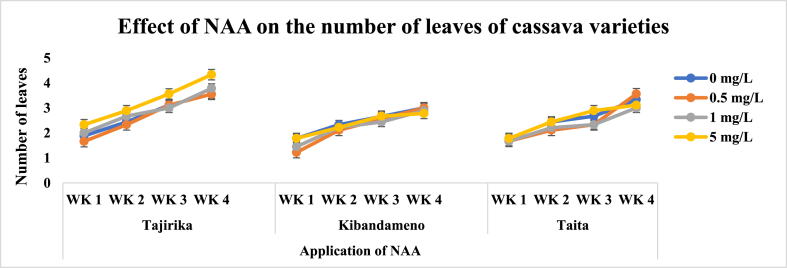
Effect of application of NAA in MS media on the number of leaves of Tajirika, Kibandameno and Taita cultivars. WK represent Weeks.

**Fig. 6:**
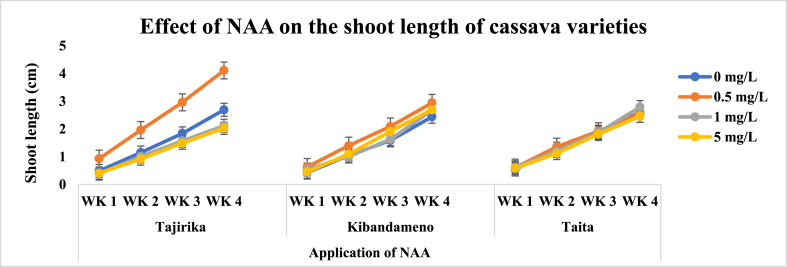
Effect of application of NAA in MS media on the shoot length of Tajirika, Kibandameno and Taita cultivars. WK represent Weeks.

There were significantly (p < 0.05) the highest number of leaves in Tajirika cultivar explant cultured in MS media supplemented with 5 mg/L of NAA in the third and fourth weeks. In the four weeks, Tajirika cultivar explants cultured in MS media supplemented with 0.5 mg/L of NAA had significantly (p < 0.05) the longest shoot length. Both Kibandameno and *Taita* cultivar explants cultured in MS media supplemented with or without NAA had no significant difference in their number of leaves and shoot lengths (Fig. 5, Fig. 6).

### 3.4 Effect of BAP and NAA on rooting of the three cassava cultivars used

**Fig. 7:**
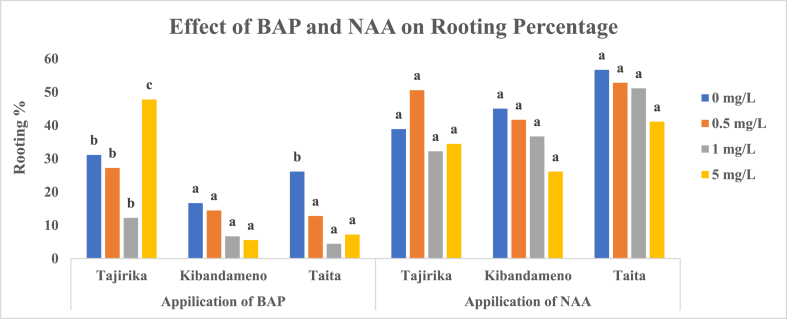
Effect of BAP and NAA in MS media on the rooting percentage of Tajirika, Kibandameno and Taita cultivars. Means in the same variety's bars having the same letter are not significantly different at P < 0.05.

**Fig. 8:**
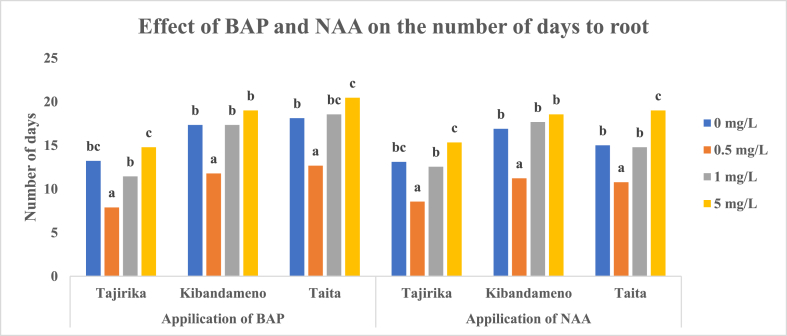
Effect of BAP and NAA in MS media on the number of days to root on Tajirika, Kibandameno and Taita cultivars. Means in the same variety's bars having the same letter are not significantly different at P < 0.05.

In Tajirika cultivar explants, significantly (p < 0.05) highest rooting of 48% was recorded in MS media supplemented in 5 mg/L of BAP whereas the least rooting of 12% was recorded when supplemented with 1 mg/L of BAP. In *Taita* cultivar, its explants cultured in MS media alone had significantly (p < 0.05) the highest rooting of 26% whereas MS media supplemented with BAP had less than 12% rooting. Kibandameno cultivar explants that were cultured in MS media with or without BAP had 5–17% rooting. Kibandameno and *Taita* cultivar explants cultured in MS media supplemented with BAP had the least rooting percentage, fortunately, took significantly (p < 0.05) fewer days (7–12 days) to root than other treatments (Fig. 7, Fig. 8).

In Tajirika, Kibandameno and *Taita* cultivar explants cultured in MS media supplemented with or without NAA had no significant difference in their rooting percentage which was 37–50%. However, significantly (p < 0.05) fewer days - 8, 11 and 10 days – were recorded in Tajirika, Kibandameno and *Taita* cultivar explants, respectively, cultured in MS media supplemented with 0.5 mg/L of NAA (Fig. 7, Fig. 8).

**Fig. 9:**
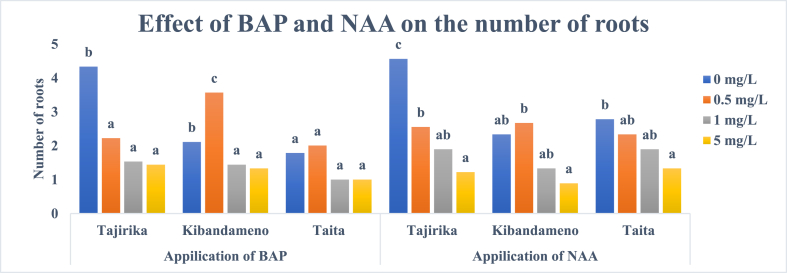
Effect of BAP and NAA in MS media on the number of roots in Tajirika, Kibandameno and Taita cultivars. Means in the same variety's bars having the same letter are not significantly different at P < 0.05.

Among the three cassava cultivars used, only Kibandameno explants cultured in MS media supplemented with 0.5 mg/L of BAP had significantly (p < 0.05) highest number of roots than other BAP supplementations. An increase in BAP concentration from 0.5 to 5 mg/L in MS media decreases the number of roots in all the cassava cultivars. Tajirika cultivar explants cultured in MS media without BAP supplementation had significantly (p < 0.05) twice the number of roots than in those treatments with BAP supplementation (Fig. 9).

Similarly, an increase in the concentration of NAA from 0.5 to 5 mg/L in MS media, decreased the number of roots in the three cassava cultivars used. All three cassava cultivars explants had about 3 roots in MS media supplemented with 0.5 mg/L of NAA but reduce to about 1 root when supplemented by 5 mg/L of NAA. Tajirika cultivar explant cultured in MS media alone had significantly (p < 0.05) the highest number of roots, about 5 roots (Fig. 9).

### 3.5 Effect of BAP and NAA on root length of the cassava cultivars used

**Fig. 10:**
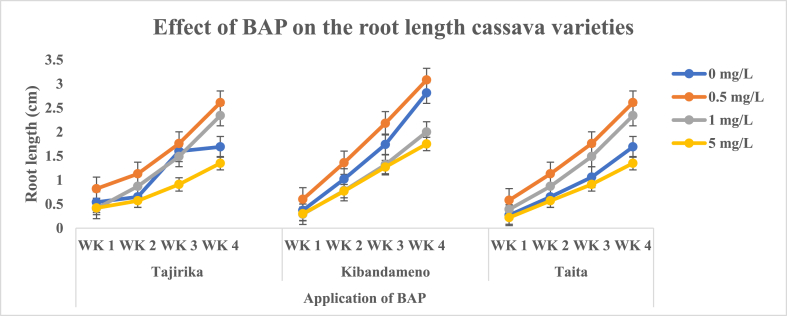
Effect of application of BAP in MS media on the root length of Tajirika, Kibandameno and Taita cultivars. WK represents Weeks.

The root lengths increased over the four weeks across all the cassava cultivars (Picture 1). An increase in the concentration of BAP from 0.5 to 5 mg/L in MS media had a decrease in the root length of the three cassava cultivars. In the three cassava cultivars, significantly (p < 0.05) the longest root lengths were recorded in explants cultured in MS media alone and supplemented with 0.5 mg/L of BAP whereas the least were recorded in MS media supplemented with 5 mg/L of BAP. The longest root of 2–3 cm was obtained in the third and fourth week in MS media alone or supplemented with 0.5 mg/L of BAP (Fig. 10).

**Fig. 11:**
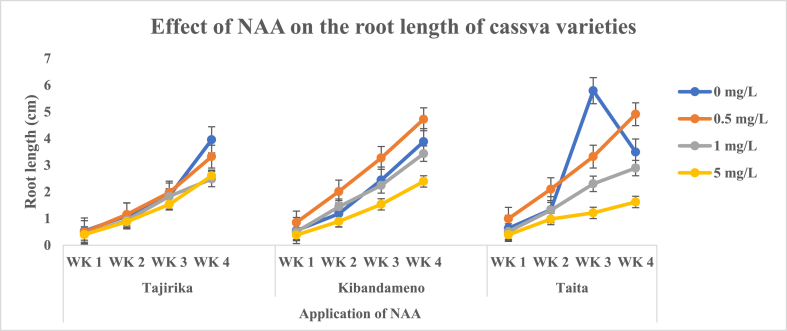
Effect of application of NAA in MS media on the root lengths of Tajirika, Kibandameno and Taita cultivars. WK represents Weeks.

In the three cassava cultivars, significant differences in root lengths were observed in the third and fourth weeks on explants cultured in MS media alone or supplemented with 0.5–5 mg/L of NAA. Kibandameno and *Taita* cultivars explants cultured in MS media alone and supplemented with 0.5 mg/L of NAA had significantly (p < 0.05) longest root lengths in the third and fourth weeks. Tajirika cultivar explants cultured in MS media alone had significantly (p < 0.05) highest root length than those supplemented with 0.5–5 mg/L of NAA in the third and fourth weeks (Fig. 11).

**Picture 1:**
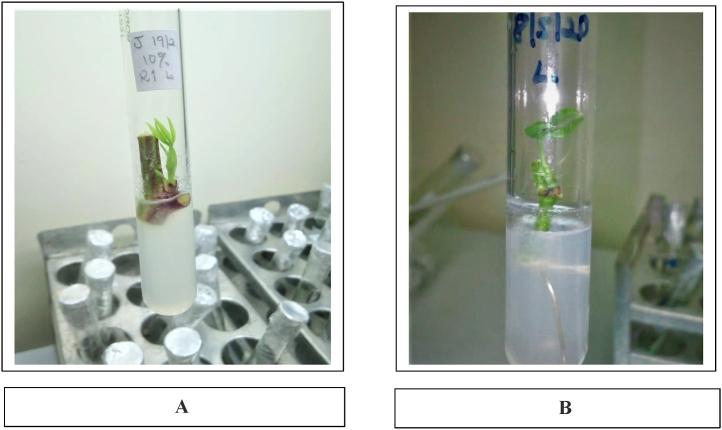
1A showed shooting of cassava explant in MS media, 1B showed a cassava explant with shoots and roots grown in MS media.

## 4 Discussion

### 4.1 The role of tissue culture technology in the mass propagation of cassava cultivars

Propagation of cassava is conventionally through cuttings that are recycled from previously harvested crops. Occasionally, the majority of cassava cultivars are infested by virus vectors responsible for cassava brown streak disease and cassava mosaic disease [[19], [20], [21]]. A study by Refs. [22,23] reported several cassava cultivars that are either resistant or tolerant to chronic pests and diseases but the majority run short on their availability of planting materials. Majority of farmers had developed a tendency on relying and planting cassava landraces owing to a preference for traits such as sweet taste that run shot in improved cassava cultivars that are characterized as being pests and disease-resistant [24]. Ref. [19] reported that cassava landraces which majority are susceptible to pests and diseases, had high yields when grown at low disease-pressure sites. Therefore, cassava landraces stand out for exploitation through phytosanitary control measures such as the generation of healthy planting material – for instance through tissue culture technology – followed by planting in low disease pressure sites. Tissue culture technology is a cost-effective alternative with the potential of mass propagation of disease-free and preferred cassava landraces. Furthermore, this tissue culture technology is a key component in this process to regenerate and certified indexed cassava planting plantlets that can be made available to farmers as hardened or for hardening as mother plants. According to Ref. [25], disease-free tissue-based mother plants facilitate the provision of healthy cassava planting materials and minimize the possibility of seed-borne diseases that negatively affected cassava yields. In consideration for mass propagation, at least 50% of tissue-based plantlets should be targeted for further hardening and to achieve this, optimization of MS media with plant growth regulators and modifications on sterilants are necessary.

### 4.2 Effects of sodium hypochlorite and 70% ethanol on initiation of cassava cultivars

Contamination with microorganisms hinders the growth of micropropagated plants because they use nutrients from the media as their energy source. According to Ref. [26], plant propagation through tissue culture is often limited by the presence of microorganism contaminations either on the explants or in the culturing media. Microorganisms are either exogenous or endogenous and have the potential to negatively affect the growth and development of micropropagated plants. For instance, endogenous bacteria and fungi potentially inhibit the healthy growth of plant roots without exhibiting the associated disease symptoms [27,28] whereas the endophytic bacteria or fungi normally generate phytotoxins responsible for plant deterioration [29]. Many studies have demonstrated the importance and success of sterilizing the explants with NaOCl and ethanol [30,31]. For example [32], showed a successful elimination of exogenous bacteria on plant surfaces using ethanol and sodium hypochlorite. Notably, endophytic microorganisms are not fully eliminated by the use of only surface sterilization but by further incorporation with antibiotics [33]. reported the control of latent contamination upon explants' surface sterilization followed by culturing in MS media with antibiotics. In this study, the success of the surface sterilants was based on the initiation percentages of the cassava cultivars and not on establishing the level of reduction of either exogenous or endogenous microorganisms. This study had an improved cassava cultivar – Tajirika – possibly with resistance or tolerance traits against diseases and pests and two landraces -Kibandameno and *Taita* – that were considered highly susceptible to pests and diseases. Therefore, the characteristics of these two cultivars, improved and landraces do not adequately justify more or less the presence of either exogenous or endogenous microorganisms when used for explants. In other instances, the sources of explants – mother plants – under high disease pressure can partly contribute to a high of either exogenous or endogenous, however, its initiation outcomes upon surface sterilization cultured in MS media with or without antibiotics remain unclear.

Effective sterilization facilitates subsequent developmental activities in cultured plants by inhibiting contaminations [34]. On the other hand, a decrease in shoot and root proliferation is eminent owing to latent infections that consequently result in an increased rate of tissue necrosis and culture mortality. For example, in tissue culture cassava, there was a 47% loss due to microbial contaminations [35] whereas it was 40–60% 47% among bananas [36]. This study has revealed that sterilization is paramount and sterilants at different concentrations and exposure times affect the cassava cultivars' explants differently at initiation. Sterilization of the three cassava cultivar explants with 15% NaOCl and exposure to 70% ethanol for 5 min had the least initiation. Low initiation could be due to ethanol, a potent sterilizing agent which is highly toxic, especially at high concentrations [37]. Sodium hypochlorite works by denaturing proteins in microorganisms and must reach the target site by passing through the bacteria's outer membrane [38]. On the other hand, an increase in exposure time to 70% ethanol reduced the initiation of explants despite sterilizing using the optimal concentration of NaOCl for the three cassava cultivars. Ethanol is a powerful sterilizing agent but also extremely phytotoxic. Therefore, the explant is typically exposed to it for only a few seconds or minutes. Ethanol is usually combined with sodium hypochlorite for effective sterilization [39]. Prudent selection of explants from the healthy parent plants coupled with an effective surface sterilization method should be the goal in avoiding culture contamination [40,41].

Studies have shown that peroxidase and polyphenol oxidase are protective enzymes against pest attacks in plants [42,43]. Tajirika cultivar is resistant to pests and diseases which could be partly attributed to its improvement, particularly on leaves through lignification. Enhancement of these enzymes confers resistance. Therefore, Tajirika-cultivar had the potential of withstanding the surface sterilizing agents at high concentrations and for a long exposure since it may have lignified and strengthened plant cells resistant to biodegradation by virtue of being resistant to pests and diseases [44,45]. On the other hand, Kibandameno and *Taita* landraces that are susceptible to pests and diseases possibly due to a lack of lignin, had a low initiation percentage when using surface sterilizing agents at high concentrations and for a long exposure [46]. showed a 40% survival in the “Sree Padmanabha (MNga-1)” cassava cultivar that was resistant to cassava mosaic disease following its surface sterilization with 3% NaOCl for 10–15 min but about 80% with 0.08–0.1 HgCl_2_ for 7 min. Numerous studies have demonstrated the use of different sterilizing agents for varying duration to optimize for use in different cassava cultivars [31,47,48]. Besides [49], demonstrated the synthesis of phytohormones' potential to improve plant growth and development by incorporating plant growth-promoting microbes in the culturing medium. From the few studies that inoculated plant growth-promoting microbes [50,51], still, the presence of microorganisms is perceived as negative considering the *in vitro* environment whereby more effort is on the detection and elimination of microorganisms [27].

### 4.3 Effect of plant growth regulators in cassava explants

The success of tissue culture has long been enjoyed following the potential of its culture medium to profoundly respond to modifications to stimulate the growth of different plants. Key ingredients in plant tissue culture media are inorganic nutrients, carbon sources, organic supplements, solidifying agents whereas plant growth regulators are mostly incorporated to enhance growth and development in different crops. Though optimal inorganic nutrients in plant tissue culture medium are considered responsible for enhancing growth and morphogenesis, however [52], confirmed the occurrence of physiological disorders, for instance, hyperhydricity, hooked leaves and fasciation and shoot tip necrosis due to an imbalance of inorganic nutrients concentrations. Notably, this study assessed initiation following surface sterilization which was inclusive of undifferentiated leaves and stems in the form of a green protrusion and the developed shoot and leaves after 14 days. Of the explants that responded in growth, about 50% developed shoots and leaves. This suggests that further developmental stages of cultured cassava explants need facilitation through plant growth regulators. In other consideration, the presence of exogenous and endogenous microorganisms could partly create an imbalance of inorganic nutrients which were considered responsible for morpho-physiological disorders by Ref. [53]. Other studies had considered the incorporation of silicon in culturing medium to improve the growth and development of various plants [[54], [55], [56]], therefore, the benefits of silicon in cassava micropropagation remains unexplored.

Plant culturing media had been extensively explored in its responses to supplementation with PGRs that have the potential of adapting a plant to enhance or retards growth and development [[57], [58], [59]]. The supplementation of MS media with 5 mg/L of BAP had the highest shooting percentage in Tajirika and *Taita* cultivars. Supplementation of MS media with either 0.5 or 1 mg/L BAP had the least shooting but took considerably the shortest time. On the other hand, supplementation of MS media with NAA had a high shooting percentage but took a long time. In rooting, PGRs especially NAA at 0.5 mg/L in MS media exhibited a profound increase in rooting to about 50% within the shortest time. Therefore, this study's findings further pointed out the consideration of the highest shooting and rooting percentage within the shortest time, following the addition of PGRs for mass propagation of cassava plantlets. For instance, as per this study's findings, without considering the time to shoot and root, it can be argued that there is no need for the addition of PGRs at the initiation stage. Vice versa, in considering the time for at least 50% shooting and rooting, the rationale of the need for PGRs supplementation in MS media is reasonable. Fortunately, or not, some studies on cassava micropropagation optimize tissue culture protocols to suit economical cost efficiency and in most instances, time seemed irrelevant.

Over time, there is perpetual use of conventional cuttings in cassava, from mother stock, perhaps in a continuation or preference of particular traits. Of course, this study used nodal with at least a bud as an explant, rationale considered to regenerate from the pre-existing meristems which are analogous to the field cuttings since it was not through dedifferentiation of tissues. The nodal explants – meristem micropropagation – were considered to remain faithful to the phenotype of the parental plants, including cassava, but [14] showed a clear divergence between field-grown cuttings and those micropropagated through meristem culture. Furthermore, 102 sites in the cassava genome were identified to be highly impacted through the meristem micropropagation, possibly due to stress-induced conditions during *in vitro* growth [14,60]. Though this study did not assess the possibility of epigenetic differences in its three varieties, more commonly preferred for their traits, it recommended its incorporation in other protocols and further explore the implications of those possible noted differences, not only in cassava.

## 5 Conclusion

In developing a tissue culture protocol, the effect of sterilants and plant growth regulators on *in vitro* micropropagation of cassava was optimized. The optimal surface sterilization protocol for Tajirika was 10% NaOCl sprayed with 70% ethanol for 20 s whereas for Kibandameno and *Taita* cultivars was 5% NaOCl with 70% ethanol sprayed for 20 s. Supplementing MS media with 5 mg/L BAP enhanced shooting in Tajirika (85%) in 4–5 days whereas MS alone was effective in Kibandameno and Taita cultivars at 86 and 91%, respectively, in 7–9 days. Supplementing MS with NAA promoted rooting of the plantlets with reduced period for rooting in 0.5mg/L in MS. Further experimentation can be done to observe what happens with concentrations between 0 and 0.5 mg/L NAA in MS for an optimal protocol for use at sub-culturing stage. Supplementation of MS media with NAA reduced days to rooting in Tajirika (7–9 days) compared to 11–20 days for the cassava landraces but it increased the root length in Kibandameno and *Taita* cultivars to 6 cm. This protocol can be adopted for use in regenerating cassava plantlets over a short time to rapidly multiply clean cassava planting materials required by small-scale farmers.

## Author contribution statement

PATRICK CLAY KIDASI: Conceived and designed the experiments; Performed the experiments; Analyzed and interpreted the data; Contributed reagents, materials, analysis tools or data; Wrote the paper. DORA CHAO KILALO: AGNES WAKESHO MWANG'OMBE: Conceived and designed the experiments; Contributed reagents, materials, analysis tools or data.

## Data availability statement

Data will be made available on request.

## Declaration of competing interest

The authors declare that they have no known competing financial interests or personal relationships that could have appeared to influence the work reported in this paper.
